# Insights into the Relationship between Toll Like Receptors and Gamma Delta T Cell Responses

**DOI:** 10.3389/fimmu.2014.00366

**Published:** 2014-07-31

**Authors:** Asif Amin Dar, Rushikesh Sudam Patil, Shubhada Vivek Chiplunkar

**Affiliations:** ^1^Chiplunkar Laboratory, Advanced Centre for Treatment, Research and Education in Cancer (ACTREC), Tata Memorial Centre, Navi Mumbai, India

**Keywords:** immunotherapy, γδ T cells, toll like receptors, tumors, dendritic cells

## Abstract

The tumor microenvironment is an important aspect of cancer biology that contributes to tumor initiation, tumor progression and responses to therapy. The composition and characteristics of the tumor microenvironment vary widely and are important in determining the anti-tumor immune response. Successful immunization requires activation of both innate and adaptive immunity. Generally, immune system is compromised in patients with cancer due to immune suppression, loss of tumor antigen expression and dysfunction of antigen presenting cells (APC). Thus, therapeutic immunization leading to cancer regression remains a significant challenge. Certain cells of the immune system, including dendritic cells (DCs) and gamma delta (γδ) T cells are capable of driving potent anti-tumor responses. The property of MHC-unrestricted cytotoxicity, high potential of cytokine release, tissue tropism and early activation in infections and malignant disease makes γδ T cells as an emerging candidate for immunotherapy. Various strategies are being developed to enhance anti-tumor immune responses of γδ T cells and DCs one of them is the use of novel adjuvants like toll like receptors (TLR) agonists, which enhance γδ T cell function directly or through DC activation, which has ability to prime γδ T cells. TLR agonists are being used clinically either alone or in combination with tumor antigens and has shown initial success in both enhancing immune responses and eliciting anti-tumor activity. TLR activated γδ T cells and DCs nurture each other’s activation. This provides a potent base for first line of defense and manipulation of the adaptive response against pathogens and cancer. The available data provides a strong rationale for initiating combinatorial therapy for the treatment of diseases and this review will summarize the application of adjuvants (TLRs) for boosting immune response of γδ T cells to treat cancer and infectious diseases and their use in combinatorial therapy.

## Introduction

Innate and adaptive immune responses are sentinels of host against the diverse repertoire of infectious agents (viruses and bacteria) and cancer. Both components of immune system identify invading microorganisms or damaged tissues as non-self and activate immune responses to eliminate them. Efficient immune responses depend upon how close an interaction is between the innate and adaptive immune system. γδ T cells and toll like receptors (TLR) serve as an important link between the innate and adaptive immune responses (1–3). Extensive studies have suggested that γδ T cells play important roles in host defense against microbial infections, tumorigenesis, immunoregulation and development of autoimmunity. γδ T cells also have several innate cell-like characters that allow their early and rapid activation following recognition of cellular stress and infection (4, 5). However to accomplish these functions, γδ T cells use both the T cell receptor (TCR) and additional activating receptors (notably NKG2D, NOTCH, and TLR) to respond to stress-induced ligands and infection. γδ T cells express TLRs and modulate early immune responses against different pathogens (6). In this review, we summarize and discuss some of the recent advances of the γδ T cell biology and how direct control of γδ T lymphocyte function and activation is monitored by TLR receptors and ligands. The review highlights involvement of TLR signaling in γδ T cell functions and their implications in harnessing γδ T cells for cancer immunotherapy.

## γδ T Cells, Anatomical Distribution and Antigenic Diversity

Based on the type of TCR they express, T lymphocytes can be divided into two major subsets, αβ and γδ T cells. γδ T cell represents a small subset of T lymphocytes (1–10%) in peripheral blood. While in anatomical locations like small intestine, γδ T cells comprise a major bulk of T cells (25–60% in human gut) (7). γδ T cells are the first T cells to appear in thymus during T cell ontogeny in every vertebrate (8), which suggests that their primary contribution could be neonatal protection because at this point conventional αβ T cell responses are severely functionally impaired and DCs are immature (9). In neonates, the Vδ2^+^ cells derived from human cord blood showed early signs of activation. These cells secrete IFN-γ and express perforin after short-term *in vitro* stimulation (10). In comparison to the neonate derived αβ T cells of peripheral blood, γδ T cell subset produces copious amount of IFN-γ and are precociously active (11). Hence, γδ T cells are well engaged in newborns to contribute to immune-protection, immune-regulation and compensate for impaired αβ T cell compartment.

γδ T cells are unconventional CD3^+^ T cells and differ from the conventional αβ T cells in their biology and function (Table 1). Although a sizeable fraction of γδ T cells in the intraepithelial lymphocyte compartments of human and mice are CD8αα^+^ but the peripheral blood γδ T cells are predominantly double negative (CD4^−^CD8^−^) T cells. The absence of CD4 or CD8 expression on majority of the circulating γδ T cells is well in line with the fact that antigen recognition is not MHC restricted (12, 13). Crystal structure analysis of the γδ TCR revealed that γδ TCR is highly variable in length resembling immuno-globulins (Ig) more than the αβ TCR. The antigen recognition property of γδ T cells is fundamentally different from αβ T cells but similar to antigen–antibody binding, which is more likely to occur independent of MHC cross presentation (14). However, recently butyrophilin BTN3A1, a non-polymorphic ubiquitously expressed molecule was identified as an antigen presenting molecule of Vγ9Vδ2 T cells. Soluble BTN3A1 binds (Isopentenyl diphosphate) IPP and (E)-4-hydroxy-3-methyl-but-2-enyl diphosphate (HMBPP) with different affinities in 1:1 ratio to stimulate γδ T cells (15).

**Table 1:** **Comparison between αβ and γδ T cells**.

S.No.	αβ T cells	γδ T cells
1	Constitutes about 65–70% of total PBMCs	Constitutes about 1–10% of total PBMCs
2	Recognize the processed peptide antigen with the help antigen presenting molecule MHC1 and MHC II	Do not show MHC restriction but may require the antigen presenting molecule Butyrophilin 3A1 molecule
3	Express either CD8^+^ or CD4^+^	Mostly double negative, murine intestinal IELs may be CD8αα^+^
4	TCR junctional diversity is very diverse	TCR junctional diversity is small
5	Do not show tissue tropism	Show tissue tropism
6	αβ T Cells response is late	γδ T cells respond earlier
7	Regulatory phenotype is attributed to CD4^+^CD25^+^ T cells	Regulatory phenotype is attributable to various subsets, including murine Vγ5^+^ DETCs and human Vγ1^+^ peripheral cells

The important feature of γδ T cells is their tropism to epithelial tissues. With respect to anatomical localization, γδ T cell population can be divided into two groups: lymphoid-homing γδ T cells that can be primed in the circulation and clonally expand in a conventional “adaptive” manner; and innate-like cells that respond rapidly and at a relatively high frequency in many tissue sites. Migration and anatomical localization of T lymphocytes is crucial for their antigen specificity and maintaining homeostasis in the mammalian immune system. Although γδ T cells are well represented among peripheral blood mononuclear cells (PBMC) and in afferent and efferent lymph, they are rarely found in lymph node parenchyma, spleen, Peyer’s patches and thymus. Moreover, unlike αβ T cells, splenic γδ T cells, if present, are not confined to the lymphoid areas (the white pulp) but are also found throughout the red pulp of spleen and marginal zones of cell trafficking (16). γδ T cells are abundantly present in the epithelia of skin, genital and intestinal tract (17). In the small intestines of humans, mice, chickens and cattle, γδ T cells comprise a substantial fraction of intestinal intraepithelial lymphocytes (IELs); in mice γδ^+^ IELs constitute 50–60% of the IEL pool (18–20). The epidermal γδ^+^ IELs of mice and cattle (but not humans) have a marked dendritic morphology and are hence known as dendritic epidermal T cells (DETCs) (21). DETCs are maintained at steady state in normal adult murine skin but on activation execute specialized functions like tissue repair (22). DETCs also maintain keratinocyte homeostasis, which along with Langerhan cells forms its neighborhood (23). Under pathological conditions, γδ T cells quickly expand and infiltrate into lymphoid compartments and other tissues.

Another striking difference between αβ and γδ T cells is the range of antigens or ligands that are recognized by the respective TCRs. Unlike αβ T cells, which recognize protein antigen processed inside the cell and presented by MHC molecules, γδ T cells recognize antigens like B cells as revealed by structural and functional studies (24).γδ T cells can respond to a variety of stimuli irrespective of their molecular or genetic nature. In mice, the non-classical MHC class I molecules T10 and T22 are recognized by γδ T cells (25–28). Similar to T10 and T20, murine class II MHC (IA) antigens IE and IA are identified to act as ligands for γδ T cell clones (29, 30). In addition, herpes glycoprotein GI-reactive γδ T cell clones protect mice from herpes simplex virus (HSV) induced lethal encephalitis (31, 32). γδ TCRs can also bind to an algal molecule, phycoerythrin inducing upregulation of CD44 and downregulation of CD62L in γδ T cells (33). B6 murine splenic and hepatic γδ T cells respond to cardiolipin (bacterial cell-wall phospholipid and endogenous component of mitochondria) presented by CD1d molecules (34). Insulin derived peptide B:9–23 is also recognized by the γδ T cell clones derived from non-obese diabetic mice (NOD mice) (35). SKINT1, a mouse immunoglobulin superfamily member, bears structural similarity to human CD277 (butyrophilin 3A1) and is expressed by medullary thymic epithelial cells (mTECs) and keratinocytes that is crucial for the development of Vγ5Vδ1^+^ DETCs (36).

In humans, majority of γδ T cells express a rearranged T cell receptor (TCR) composed of Vγ9 and Vδ2 domains; thus, this population is referred to as Vγ9Vδ2. The Vγ9Vδ2 T cells recognize self and microbial phosphorylated metabolites generated in eukaryotic mevalonate pathway and in the microbial 2-C-methyl-derythritol 4-phosphate (MEP) pathway (37). Initially, it was reported that the non-peptidic ligands isolated from mycobacterial cell lysates were stimulatory for Vγ9Vδ2 T cell clones. Later, IPP, an intermediate metabolite of the mevalonate pathway, was isolated and identified as a stimulatory molecule. Characterization of the microbial antigens recognized by human γδ T cells predicted that these are non-proteinaceous in nature and have critical phosphate residues (37, 38). Subsequent studies, conducted with *M. tuberculosis*, identified HMBPP, an intermediate metabolite of the MEP pathway, as a strong agonist of γδ TCR. The measured potencies of IPP and HMBPP show an enormous difference. The ED50 of IPP is ~20 μM, whereas that of HMBPP is ~70 pM, i.e., more than 105 times lower (38).

Another stimulatory molecule is *Staphylococcus aureus* enterotoxin A (SEA) that directly interacts with the TCR Vγ9 chain independently of the paired Vδ chain. The mechanism of recognition of this superantigen is different from that of phosphorylated metabolites and requires the interaction with MHC class II molecules. γδ T cells kill target cells and release cytokines upon interaction with SEA but do not proliferate (39).

Recently, the TCR from a γδ T cell clone derived from a cytomegalovirus (CMV)-infected transplant patient was shown to directly bind to endothelial protein C receptor (EPCR), which is a lipid carrier with a similar structure to CD1, showing again that γδ TCR engagement is cargo independent (40). ATP F1 synthase has been identified as stimulatory ligand of the TCR Vγ9Vδ2. ATP F1 synthase is an intracellular protein complex involved in ATP generation. However, optimal responses of Vγ9Vδ2 T cells by tumor target cell lines expressing F1-ATPase requires apolipoprotein A1. A monoclonal antibody interacting with apolipoprotein A1 was shown to inhibit TCR γδ activation as it disrupted the trimolecular complex of ApoA1, ATP F1 synthase, and γδ TCR required for optimal response (41).

The second major population of human γδ T cells utilizes the Vδ1 chain, which pairs with a variety of Vγ chains. This subset of Vδ1^+^ T cells is mainly found in tissues and is activated by CD1c and CD1d-expressing cells. The group 1 CD1 molecules have ability to present lipid A to human γδ T cells. The human γδ T cells also recognize the related group 2 CD1 molecule as CD1d/lipid complex. Phosphatidyl ethanol amine (PE), a phospholipid, activates γδ T cells in a CD1d manner dependent suggesting its CD1d restricted recognition (42). In addition, some populations of γδ T cells in normal human PBMCs also recognize lipid molecules such as cardiolipin (a marker of damaged mitochondria), sulfatide (a myelin glycosphingolipid), or α-galactosylceramide (α-GalCer) in association with CD1d, which are noted ligands of natural killer T (NKT) cells (34, 43–45). Human γδ T cells also recognize the stress-induced MHC class I-related MICA/MICB molecules and the UL16-binding proteins that are upregulated on malignant or stressed cells (46–48). Heat shock proteins (HSPs) expressed on the cell membrane play an important role in cancer immunity. Hsp60 expressed on oral tumors act as ligand for Vγ9Vδ2 T cells (49, 50). Hsp60 and Hsp70 expressing human oral and esophageal tumors are lysed by Vγ9Vδ2 T cells (49–51). Hsp72 expressing neutrophils were rapidly killed by γδ T cells through direct cell to cell contact, indicating that hsp72 expression on cell surface pre-disposes inflamed neutrophils to killing by γδ T cells (52). In Another study, hsp90 expression on EBV infected B cells rapidly promoted γδ T cell proliferation (53). This confirms that γδ T cells recognize qualitatively distinct antigens, which are profoundly regulated by their anatomical localization.

## Co-Receptors and γδ T Cell Activation

Most γδ T cells respond to non-peptidic antigens even in the absence of antigen presenting cells (APCs). However, the presence of APCs can greatly enhance the γδ T cell response (54). This suggests that accessory molecules/receptors may be involved in effector functions of these cells. Some of important co-receptors used by γδ T cells include NOTCH, NKG2D, and TLR (55).

Our study has identified Notch as an additional signal contributing to antigen specific effector functions of γδ T cells. We have shown that γδ T cells express Notch1 and Notch2 at both mRNA and protein level. Inhibition of Notch signaling in anti-CD3 MAb stimulated γδ T cells resulted in marked decrease in proliferation, cytotoxic potential, and cytokine production by γδ T cells confirming the involvement of Notch signaling in regulating antigen specific responses of γδ T cells (55).

γδ T cells express NKG2D on their cell surface resulting in their activation. Treatment of PBMC with immobilized NKG2D-specific mAb or NKG2D ligand MHC class I related protein A (MICA) resulted in the up-regulation of CD69 and CD25 on Vγ9Vδ2. Furthermore, NKG2D increased the production of TNF-alpha and release of cytolytic granules by Vγ9Vδ2 T cells (56). Later, it was shown that the protein kinase C transduction pathway as a main regulator of the NKG2D-mediated costimulation of anti-tumor Vγ9Vδ2 T cell cytolytic response (57).

TLR agonists are also known to trigger the early activation and the IFN-γ secretion by Vγ9Vδ2T cells (58). TLR ligands indirectly increase the anti-tumoricidal activity of Vγ9Vδ2T cells (59). In this review, we will focus on TLR as an additional co-receptor modulating the function of immune cells with special focus on γδ T cells.

## Toll Like Receptor and Immune Cells

The immune system functions in anti-microbial defense by recognizing groups of molecules unique to microorganisms (60). These unique microbial molecules are called pathogen-associated molecular patterns (PAMPs) and are recognized by a family of cellular receptors called pattern recognition receptors (PRRs) (61). TLRs along with retinoic acid-inducible gene (RIG)-I-like receptors (RLRs) and nucleotide-binding oligomerization domain (NOD)-like receptor (NLRs) are prototype PPRs, which recognize pathogen-associated molecular patterns (PAMPs) from microorganisms or danger-associated molecular patterns (DAMPs) from damaged tissues (62). Recognition of PAMPs by TLRs trigger release of inflammatory cytokines and type 1 interferon’s (IFN) for host defense (60, 63–65). The adaptive immune system, on the other hand, is responsible for elimination of pathogens in the late phase of infection and in the generation of immunological memory mediated by B and T cells (66).

TLRs derived their name from *Drosophila melanogaster* Toll protein based on their homology (67). In mammals, till date 13 members of TLR family has been identified (63, 68–71). TLR1-9 is conserved in humans and mice while TLR10 is non-functional in mice because of a retroviral insertion while TLR11-13 is lost from the human genome. The first TLR identified was TLR4 and recognizes bacterial lipopolysaccharide (LPS) from Gram-negative bacteria (67, 72, 73). TLRs are classified into several groups based on the types of PAMPs they recognize. TLR1, 2, 4 and 6 recognize lipids whereas the highly related TLR7, TLR8 and TLR9 recognize nucleic acids. Murine TLR11 recognizes a protozoan derived profilin-like protein while TLR13 recognizes *Vesicular stomatitis virus* (63). TLRs are localized in the distinct cellular compartments, for example; TLR1, TLR2, TLR4, TLR5, TLR6, and TLR11 are expressed on the cell surface whereas TLR3, TLR7, TLR8 TLR9, TLR11, TLR12 and TLR13 are expressed in intracellular vesicles such as the endosome and ER. The intracellular TLRs are transported to the intracellular vesicles via UNC93B1, a trans-membrane protein, which is localized in the ER of the cell (70, 71, 74–77). TLR family receptors have a common structural architecture. TLRs are type I integral membrane glycoproteins characterized by multiple extracellular leucine-rich repeats (LRRs) and a single intracellular Toll/interleukin-1 (IL-1) receptor (TIR). TLRs mostly form homo-dimers with a few exceptions, which form heterodimers to trigger a signal. For example, TLR2 forms heterodimers with TLR1 or TLR6 enabling differential recognition of lipopeptides. The TIR domain of TLRs is required for the interaction and recruitment of various adaptor molecules to activate downstream signaling pathway. After recognizing PAMPs, TLRs activate intracellular signaling pathways that lead to the induction of inflammatory cytokine genes such as TNF-α, IL-6, IL-1β and IL-12 through the recruitment of adaptors such as MyD88, TRIF, TRAM, TIRAP and SARM1 (78). MyD88 is a universal adaptor used by all TLRs, except TLR3, to induce inflammatory pathways through activation of MAP Kinases (ERK, JNK, p38) and transcriptional factor NF-κB (63, 79). TLR3 and TLR4 use TRIF to bring activation of alternative pathway (TRIF-dependent pathway) through transcription factors IRF3 and NF-κB to induce type 1 IFN and inflammatory cytokines (80–82). TRAM selectively participates in the activation of the TRIF-dependent pathway downstream of TLR4, but not TLR3 (83, 84). TIRAP functions to recruit MyD88 leading to activation of MyD88-dependent pathway downstream of TLR2 and TLR4 (85, 86).Sterile-α- and armadillo-motif-containing protein 1 (SARM1), was shown to inhibit TRIF and is also critical for TLR-independent innate immunity (87). Thus, signaling pathways can be broadly classified as either MyD88-dependent pathway or TRIF-dependent pathway.

Hornung et al. have showed differential expression of TLR1-10 on human APCs and lymphocytes including T cells and their functional discrepancy in recognition of specific TLR ligands (88). CD4^+^ T cells express almost all TLRs at mRNA levels but may not express all as functional protein (89, 90). Moreover, they do not respond to all TLR ligands. Stimulation with TLR5, 7, or 8 agonists combined with TCR activation of CD4^+^T cells resulted in increased proliferation and production of IL-2, IL-8, IL-10, IFN-γ and TNFα (91). There are other reports as well suggesting the functional modulation of subtypes of CD4^+^ T cells by TLR ligands. The mouse Th1 but not Th2 cells responded to TLR2 agonist and resulted in enhanced proliferation and IFN-γ production independent of TCR stimulation (92). This work validated that the TLR can regulate function of CD4^+^ T cells even in absence of TCR engagement. CD4^+^CD25^+^ regulatory T cells (Tregs) express majority of TLRs with selectively higher expression of TLR2, 4, 5, 7/8, and 10 compared to CD4^+^CD25^−^ conventional T cells (93). Liu et al. showed that CD4^+^CD25^+^ regulatory T cells and CD4^+^CD25^−^ conventional T cells express TLR2 and proliferated upon stimulation with its agonist. TLR2 stimulation also led to transient loss of Treg suppressive potential through suppression of FOXP3 (94, 95). However, Tregs also express TLR5 but upon stimulation with flagellin (ligand of TLR5), do not proliferate rather showed increased suppressive capacity and enhanced expression of FOXP3 (96). These reports suggest that the suppressive function of Treg can be either enhanced or dampened by the type of TLR ligand engaged. TLR2 stimulation not only abrogates suppressive functions of CD4^+^ Tregs but also drives naïve as well as effector Treg population toward IL17 producing Th17 phenotype (97). Th17 cells express TLR2 along with TLR6 compared to Th1 and Th2 subsets and promote Th17 differentiation upon Pam3Cys stimulation and accelerates experimental autoimmune encephalomyelitis (98). Like TLR2, TLR4 also regulate the functions of CD4^+^ T cells. In a mouse model of arthritis, mice lacking TLR2 showed enhanced histopathological scores of arthritis by a shift in T cell balance from Th2 and T regulatory cells toward pathogenic Th1 cells. TLR4, in contrast, contributes to more severe disease by modulating the Th17 cell population and IL-17 production (99, 100). Recently, Li et al. showed that high-mobility group box 1 (HMGB1) proteins decrease Treg/Th17 ratio by inhibiting FOXP3 and enhancing RORγt in CD4^+^ T cells via TLR4–IL6 axis in patients with chronic hepatitis B infections (101). This shows that HMGB1 (TLR4 ligand) act as a modulator of CD4^+^ T cells responses in chronic viral inflammation. CD4^+^ T cells also express intracellular TLRs such as TLR9 and TLR3. Both these TLRs promote T cell survival via activation of NF-κB and MAPK signaling (102). Although the effector functions of CD4^+^ T cells are regulated by TLRs but the molecular pathway involved in skewing of CD4^+^ T cell function is poorly understood.

Like CD4^+^ T cells, CD8^+^ T cells also show differential expression of TLRs with high expression of TLR3 but lower expression of TRL1,2,5,9,10 compared to CD4^+^ T cells at mRNA level. It is important to note that the expression of TLR2, TLR3 and TLR5 increases on CD8 T cells in infected tonsils compared to controls (89) indicating immune activating role of TLRs in infections. Stimulation of CD8^+^ T cells through TLR2 agonists enhances their proliferation and IFN-γ production (103, 104). It also promotes cytolytic activity of CD8^+^ T cells and enhances anti-tumor response mediated through MyD88-dependent TLR1/2 pathway (105). Recently, Mercier et al. showed that TLR2 cooperate with NOD-containing protein 1 (NOD1) to enhance TCR mediated activation and can serve as alternative co-stimulatory receptor in CD8^+^ T cells (106). CD8^+^ T cells also express intracellular TLRs such as TLR3, TLR9 which are more potent in inducing CD8^+^ T cell activation *in vivo* (107).

Natural killer (NK) cell is a vital player in innate immune system. They recognize infected and transformed cells with downregulated major histocompatibility complex (MHC) class 1 molecules. They are the primary producers of IFN-γ and are protective against infections. Unlike CD4 and CD8 T cells NK cells as well as CD56^+^CD3^+^ NKT cells constitutively express TLR 1–8 with high expression of TLR2 and 3 at mRNA level. They recognize bacterial PAMPs and respond by producing α-defensins (108–111). Human NK cells can also directly recognize *Mycobacterium bovis* via TLR2 and enhance their cytolytic activity against tumor cells (112). Tumor-associated macrophages induce NK cell IFN-γ production and cytolytic activity upon TLR engagement (113). TLRs modulate NK cell function directly or indirectly to promote antibody dependent cell mediated cytotoxicity and cross presentation of viral antigens to T lymphocytes (114, 115). This highlights that the cells of adaptive immune system do express TLRs and their function can be directly or indirectly modulated by TLR ligands.

## Activation of γδ T Cells by TLR Ligands

In 1997, the first human homolog of *Drosophila* Toll protein was cloned and characterized. It was also established that γδ T cells also express hToll mRNA (67). Purified γδ T cells were found to respond to the *E. coli* native lipid A in a TCR-independent fashion and the LPS/lipid A-reactive γδ T cells strongly expressed TLR2 mRNA. TLR2 antisense oligonucleotide inhibited the proliferation of γδ T cells in response to the native lipid A as well as the TLR2-deficient mice showed an impaired response of the γδ T cells following injection of native lipid A. These results suggest that TLR2 is involved in the activation of canonical Vγ6/Vδ1 T cells by native lipid A (116). Again, functional presence of TLR2 on Vγ2Vδ2 T cells (also known as Vγ9Vδ2 T cells) was reported when the dual stimulation of Vγ2Vδ2 T cells with anti-TCR antibody and Pam_3_Cys increased synthesis and secretion of IFN-γ and elevated the levels of CD107a expression. IFN-γ secretion and cell surface CD107a levels are markers of increased effector function in Vγ2Vδ2 T cells (117). Similarly, Bruno et al. reported that IL-23 and TLR2 co-stimulation induces IL17 expression in γδ T cells. However, TLR1 and TLR2 expression was found only on CCR6^+^ IL-17 producing murine peritoneal γδ T cells but not others. Thus, γδ T cells with innate receptor expression coupled with IL-17 production establishes them as first line of defense that can orchestrate an inflammatory response to pathogen-derived and environmental signals long before Th17 can sense the bacterial invasion (118). Pam3CSK4, TLR2 agonist was able to stimulate only splenic γδ T cell proliferation but not the dermal γδ T cells demonstrating that TLR2 signaling shows tissue tropism. (19). Furthermore, a profound change in the circulating γδ T-cell population was observed in early burn injury (24 h). These γδ T-cells showed TLR2 and TLR4 expression, priming them for TLR reactivity, However TLR expression was specific to circulatory γδ T cell subset and was transient, since it was not observed after post-injury (7 days). Transient nature of the post-burn increase in γδ T-cell TLR expression is likely to be protective to the host, most likely via regulation of inflammation and initiation of healing processes (119).Mitochondrial danger-associated molecular patterns (MTDs) induce TLR2 and TLR4 expression on γδ T cells in dose dependent manner. MTDs also induced the production of IL-1β, IL-6, IL-10, RANTES, and vascular endothelial growth factor by γδ T-cells thereby resulting in initiation of sterile inflammation leading to tissue/cellular repair (120).

Different studies have reported that γδ T cells express TLR3 (121, 122). TLR3 recognizes viral dsRNA, synthetic analogs of dsRNA, polyinosinic–polycytidylic acid [poly (I:C)] and small interfering (si) RNA. The direct stimulation of freshly isolated γδ T cells via TCR and surrogate TLR3 ligand poly (I:C) dramatically increased IFN-γ production. Addition of neutralizing anti-TLR3 mAb inhibited the co-stimulatory effect of poly (I:C), presumably by antagonizing the TLR3 signaling (122). Thus, the integrated signals of TLR3 and TCR induce a strong antiviral effector function in γδ T cells supporting the decisive role of γδ T cells in early defense against viral infection. In other study, it has been reported that γδ cells of term babies and of adults express TLR3 and TLR7 while the preterm babies have reduced levels. The greater levels of IFN-γ protein was observed in adult and cord blood cells co-stimulated with anti-CD3 and poly(I:C) whereas this was not seen in γδ T cell clones of preterm babies. Thus, reduced level of TLR3 expression by preterm-derived clones had an overt functional consequence on IFN-γ levels (11). Interestingly, a primary role of TLR3 in humans appears to mediate resistance to HSV-induced encephalitis (123). Hence, premature babies are particularly susceptible to HSV infection because of reduced levels of TLR3 on γδ T cells.

TLR4 was reported to be absent in the γδ T cells but can become functional in γδ T cells depending on localization, environmental signals, or γδ TCR usage (19, 118, 124). However, our own data has shown that TLR4 is expressed on human γδ T cells. Stimulation of γδ T cells with LPS (TLR4 ligand) increased their proliferation, IFN-γ release, and cytotoxic potential (125). DETCs lack cell surface expression of TLR4–MD2. MD-2 physically associates with TLR4 on the cell surface and is required for LPS signaling. However, TLR4–MD2 expression was upregulated when DETCs emigrated from the epidermis during cutaneous inflammation. The migration signals of DETCs may promote the TLR4–MD2 expression (126). Cairns et al. showed that late post-burn injury increased expression of TLR-4 on splenic T-cells (127). However, Martin et al. reported transient TLR-4 expression post-burn in the circulation or spleen but were specific for the γδ T-cell subset (119). Several evidences suggest that murine γδ T cells recognize LPS/LA through TLR2 or TLR4 (128, 129). Importantly activated γδ T cells, especially Vδ2 T cells, in peripheral blood cells recognize LA, a major component of LPS, via TLR4 resulting in extensive proliferation and production of IFN-γ and TNF-α *in vitro* (130). The data suggest that γδ T cells play an important role in the control of infection induced by gram negative bacteria. Reynolds et al. showed that a heterogeneous population of γδ T cells responds to LPS via TLR4 dependent manner and demonstrate the crucial and innate role of TLR4 in promoting the activation of γδ T cells, which contributes to the initiation of autoimmune inflammation (100). Another study showed the indirect role of TLR4 in HMGB–TLR4–IL-23–IL17A axis between macrophages and γδ T cells, which contribute to the accumulation of neutrophils and liver inflammation. Necrotic hepatocytes release HMGB1, a damage-associated molecule or TLR4 ligand, which increased IL-23 production of macrophages in a TLR4 dependent manner. IL-23 aids γδ T cells in liver in the generation of IL-17A, which then recruits hepatic neutrophils (131).

Human γδ T cells were found to express appreciable levels of TLR7. Costimulation with poly I:C upregulated the TLR7 expression in TCR-cross linked freshly isolated γδ T cells (124). In addition, tumor-infiltrating γδ T cells also express TLR7 (132). In case of mouse dermal γδ T cells, both TLR7 and TLR9 signaling promoted IL-17 production, which could be synergistically enhanced with the addition of IL-23 (19).

The identification of dominant γδ T cells in the total population of tumor-infiltrating lymphocytes (TILs) in renal, breast, and prostate cancer suggested that these cells might have the potent negative immune regulatory function (132, 133). The breast tumor-derived bulk γδ T cell lines and clones efficiently suppressed the proliferation and IL-2 secretion of naïve/effector T cells and inhibited DC maturation and function. Hence, their depletion or the reversal of their suppressive function could enhance anti-tumor immune responses against breast cancer. Indeed as in CD4^+^ regulatory T cells (Tregs), the immunosuppressive activity of γδ T cells could be reversed by human TLR8 ligands both *in vitro* and *in vivo*. Study revealed that MyD88, TRAF6, IKKα, IKKβ and p38α molecules in γδ1 cells were required for these cells to respond to TLR8 ligands (132, 134, 135). Table 2 shows expression and co-stimulatory effects mediated by TLR activation of γδ T cells

**Table 2:** **Expression and functions mediated by TLRs on γδ T cells**.

TLR	Functions	References
TLR 2	Recognize LPS, enhance proliferation, induce IFNγ and CD107a expression, enhance IL17 secretion, expression transiently increases after burn injury, mitochondrial danger-associated molecular patterns (MTDs) induce expression and production of IL-1β, IL-6, IL-10, RANTES, and VEGF	(19, 116–120)
TLR3	Induce IFNγ production in conjunction with TCR stimulation, resistance to HSV induced encephalitis	(11, 121–123)
TLR4	Increases proliferation, IFN-γ release, and cytotoxic potential, activation following burn injury	(100, 125, 127, 130)
TLR7/9	Upregulate upon poly I:C costimulation, promote IL-17 production	(19, 124, 132)
TLR8	Reversal of immunosuppressive activity	(132, 134, 135)

## TLRs Modulate Crosstalk between γδ T and Dendritic Cells

The functional fate of effector T cells is governed by antigen presentation and the cytokine milieu in the local environment. Dendritic cells (DCs) being professional APCs, recognize the danger signal, process it, and present it to the T lymphocytes thereby modulate adaptive immune response. γδ T cells influence the antigen presenting property of DCs. DCs pre-incubated with activated γδ T cells enhance the production of IFN-γ by alloreactive T cells in mixed lymphocyte reaction (136). Moreover, γδ T cells not only upregulated CD86 and MHC I expression on DC but themselves get activated, leading to up-regulation of CD25, CD69, and cytokine production (137). These studies showed how γδ T cell and DCs regulate each other’s function. There are reports, which have shown how γδ T cells interact with DC or *vice versa* via TLR ligands. Leslie et al. reported that stimulation with TLR ligands in γδ/DC cocultures enhanced the maturation and production of IL12p70 by DCs (138). TLR also regulate the γδ T cells and DC crosstalk in microbial context. TLR2-stimulated DCs enhanced IFN-γ production by Vδ2 T cells; conversely, phosphoantigen activated Vδ2 T cells enhanced TLR2-induced DC maturation via IFN-γ, which co-stimulated interleukin-12 (IL-12) p70 secretion by DCs (139). Further, γδ T cells stimulated with TLR7 (CL097) or TLR3 (poly I: C) agonists produce IFN-γ, TNFα and/or IL-6 thereby inducing DC maturation, which prime effector T cells against West Nile Virus (WNV) infection (140). This study confirmed that the antiviral effector immunity may be regulated by interplay of DCs, γδ T cells and TLRs. Similarly, in human’s γδ T cells and DCs regulate each other’s immunostimulatory functions. TLR3 and TLR4 ligands stimulation of human PBMCs induced a rapid and exclusive IFN-γ production by Vγ9Vδ2 subset dependent on type 1 IFN secreted by monocytic DC. TLR-induced IFN-γ response of Vγ9Vδ2 T cells led to efficient DC polarization into IL-12p70-producing cells (58). In another study, it was reported that Vδ2 cells are indirectly activated by BCG and IL-12p70 secreted by DCs. IL-12p70 production by DC is modulated by Toll like receptor 2/4 ligands from BCG and IFN-γ secreted by memory CD4 T cells (141). This study portrayed the complex interplay between cells of the innate and adaptive immune response in contributing to immunosurveillance against pathogenic infections.

## TLRs Complement Cytotoxic Potential of γδ T Cells Against Tumor Cells

γδ T cells have capability to lyse different types of tumors and tumor-derived cell lines (49, 50, 142–145). Circulating as well as tumor-infiltrating γδ T cells have the ability to produce abundant proinflammatory cytokines like IFN-γ and TNF-α, cytotoxic mediators and MHC-independent recognition of antigens, render them as important players in cancer immunotherapy (143, 145). In addition to TCR, γδ T cells use additional stimulatory co-receptors or ligands including TLRs to execute effector functions and TLR agonists are considered as adjuvants in clinical trial of cancer immunotherapy (146). Kalyan et al. even quoted that “TLR signaling may perfectly complement the anti-tumor synergy of aminobisphosponates and activated γδ T cells and this combined innate artillery could provide the necessary ammunition to topple malignancy’s stronghold on the immune system” (147). Paradoxically, TLR agonists execute dual role of enhancing immune response (148) as well as increasing invasiveness of tumor cells (149–152). Hence, the tripartite cooperation of tumor cell, TLRs, and γδ T cells should be carefully analyzed. In concordance to this, Shojaei et al. reported that Toll like receptor 3 and 7 agonists enhanced the tumor cell lysis by human γδ T cells. The enhanced capability of γδ T cells to lyse tumor cells was attributed to increased expression of CD54 and downregulation of MHC class 1 on tumor cells. Poly(I:C) treatment of pancreatic adenocarcinomas resulted in overexpression of CD54 and concomitant coculture of tumor cells with γδ T cells led to interaction between CD54 and its ligand CD11a/CD18 triggering effector function in γδ T cells. However, TLR7 surrogate ligand induced downregulation of MHC class 1 molecule on tumor cells resulting in a reduced affinity for inhibitory receptor NKG2A on γδ T cells (59). Manipulation of TLR signaling by using TLR8 agonists reversed the suppressive potential of γδ Tregs found elevated in breast cancer (132). Polysaccharide K (PSK) known for its anti-tumor and immuno-modulatory function can also activate TLR2 leading to increased secretion of IFN-γ by γδ T cells on stimulation. The cell–cell contact between γδ T cells and DC was required for optimal activation of γδ T cells. However, PSK along with anti-TCR could co-activate γδ T cells even in the absence of DC. The study confirmed that the anti-tumor effect of PSK was through activation of γδ T cells (153).

Studies from our lab have shown that the TLR signaling in γδ T cells derived from the oral cancer (OC) patients may be dysfunctional. We reported that γδ T cells from healthy individuals (HI) and OC patients express higher levels of TLR2, TLR3, TLR4, and TLR9 than in αβT cells. Higher TLR expression was observed in HI compared to OC patients. Stimulation with IL2 and TLR agonists (Pam3CSK, Poly I:C, LPS, and CpG ODN) resulted in higher proliferative response of peripheral blood lymphocytes from HI compared to OC patients. However, the role of other immune cells that may influence the TLR ligand stimulation induced activation status of lymphocytes cannot be ignored (125). Impairment in TLR expression/signaling can be viewed as a strategy employed by tumor cells to avoid immune recognition.

## TLRs and γδ T Cells in Diseases

Studies have demonstrated the protective role of γδ T cells in infection and inflammation (154–157). Inoue et al. showed that during mycobacterial infection, γδ T cells precedes the αβ T cells, indicating role of γδ T cells as first line of defense against infections (158). The conserved molecular patterns associated with pathogens are directly recognized by γδ T cells leading to rapid protective response against the danger signal. Unlike αβ TCR, γδ TCR acts as pattern recognition receptor providing advantage in anti-infection immunity by directly initiating cytotoxicity against infected cells or through production of cytokine to involve multiple immune system components to combat infection (159, 160). Activated γδ T cells through TLR3 and TLR4 ligands rescue the repressed maturation of virus-infected DCs and mount a potent antiviral response (58, 140). Malarial infection in MyD88 deficient mice resulted in impairment in CD27^−^IL-17A-producing γδ T cell without affecting the IFN-γ producing γδ T cells (161). This study specifies the role of TLR in promoting proliferation of proinflammatory γδ T cells. Another study by Martin et al. showed that IL17 producing γδ T cells express TLR1 and TLR2 and expand in response to their ligands and mount an adequate response against heat-killed *M. tuberculosis* or *C. albicans* infection (118). However, γδ T cell are also known to directly recognize the pathogen-derived molecules and mediate cytotoxic effector function either through secretion of perforin and granzyme B or by secretion of proinflammatory cytokine IL17 (162–164). The involvement of TLRs in regulating anti-microbial γδ T cell function should be investigated in depth to exploit it as a cell based therapy for infectious diseases.

## Concluding Remarks

The characteristic copious IFN-γ or IL17 secretion, MHC-independent antigen recognition, tissue tropism, and potent cytotoxicity make γδ T cells promising targets for immunotherapy. Similar to αβ T cells, γδ T cells exhibit functional and phenotypic plasticity, which influences the nature of the downstream adaptive immune response. The adoptive transfer of *ex vivo* expanded Vγ9Vδ2 T cells or *in vivo* activation of Vγ9Vδ2 T cells (phosphoantigens or amino-bisphosphonates) can be utilized as adjuvant to conventional therapies. Clinical trials of Vγ9Vδ2 T cells as immunotherapeutic agents have shown encouraging results that could be attributed to its low toxicity grade. Combinations of cellular immune-based therapies with chemotherapy and other anti-tumor agents may be of clinical benefit in the treatment of malignancies. Combinatorial treatment using, chemotherapeutic agents or bisphosphonate zoledronate (ZOL) sensitizes tumor-derived cell lines to rapid γδ T cells killing. Vγ9Vδ2 T cell triggering may be also enhanced by combining TCR stimulation with engagement of TLRs. Various TLR agonists are currently under investigation in clinical trials for their ability to orchestrate anti-tumor immunity. In one study, simultaneous use of both Imiquimod (TLR7 agonist) and CpG–ODN (TLR9 agonist) loaded onto virus like nanoparticles was found to be effective in triggering effector and memory CD8^+^ T cell response (165). Similarly, combination of γδ T cells and DCs along with nanoparticle loaded TLR agonists can be employed for developing effective immunotherapeutic strategies. The direct or indirect stimulation of γδ T cells by TLR agonists could be a strategy to optimize Th1-mediated immune responses as adjuvant in vaccines against infectious or malignant diseases.

**Figure 1:**
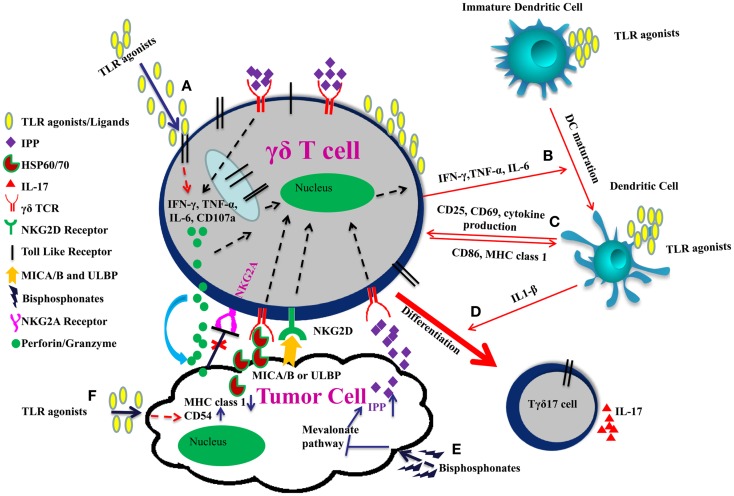
**Improving γδ T cell functions by TLRs in combinatorial therapy**. **(A)** TLR agonists induce effector function of γδ T cells through IFN-γ, TNF-α, IL-6 secretion, and increased expression of CD107a. **(B)** IFN-γ, TNF-α, and IL-6 secreted by γδ T cells and TLR agonists promote the maturation of dendritic cell. **(C)** γδ T cells upregulate CD86 and MHC I expression on DCs and are themselves activated through up-regulation of CD25, CD69, and cytokine production thereby modulating each other’s function. **(D)** Co-stimulation of γδ T cells with TLR agonists and IL-1β secreted by dendritic cells promote their polarization toward IL17 producing cells. **(E)** γδ TCR also recognizes the specific molecular patterns such as IPP, which are induced upon inhibition of mevalonate pathway by bisphosphonates. Moreover, NKG2D receptor on γδ T cells recognizes MICA/B or ULBP expressed on tumor cells. This binding enhances release of perforins and granzymes by the γδ T cells leading to tumor cell lysis. **(F)** TLR agonists act as adjuvants and can induce CD54 expression and downregulation of MHC class 1 on tumor cells. Interaction between CD54 and its ligand CD11a/CD18 trigger effector functions in γδ T cells. Downregulation of MHC class 1 molecule on tumor cells result in reduced signaling through the inhibitory receptor NKG2A on γδ T cells, which enhances the cytotoxic potential of γδ T cell.

Administration of an “immunogenic chemotherapy” (such as oxaliplatin or anthracycline or an X-ray-based regimen) or local delivery of TLR surrogates in the tumor microenvironment (which stimulate local DCs and provides a source of IL-1β) may be also instrumental in polarization of γδ TILs into IL17 producing cells. Tγδ17 cells play a crucial role in anti-microbial immunity but their role in tumor immunity remains controversial. Tγδ17 have both pro and anti-tumor properties. TLR use in combinatorial therapy, therefore, could be a double edged sword. Careful use of TLR agonists in combinatorial γδ T cell based therapy is needed to strike the balance between pro and anti-tumor effects (Figure 1).

## Conflict of Interest Statement

The authors declare that the research was conducted in the absence of any commercial or financial relationships that could be construed as a potential conflict of interest.
